# Zonal rotor centrifugation revisited: new horizons in sorting nanoparticles†

**DOI:** 10.1039/c9ra05140f

**Published:** 2019-09-02

**Authors:** Claudia Simone Plüisch, Brigitte Bössenecker, Lukas Dobler, Alexander Wittemann

**Affiliations:** Colloid Chemistry, Department of Chemistry, University of Konstanz Universitaetsstrasse 10 D-78464 Konstanz Germany alexander.wittemann@uni-konstanz.de; Particle Analysis Center, Department of Chemistry, University of Konstanz Universitaetsstrasse 10 D-78464 Konstanz Germany

## Abstract

Density gradient centrifugation is an effective method for the isolation and purification of small particles. Hollow rotors capable of hosting density gradients replace the need for centrifuge tubes and therefore allow separations at large scales. So far, zonal rotors have been used for biological separations ranging from the purification of whole cells down to serum proteins. We demonstrate that the high-resolution separation method opens up exciting perspectives apart from biology, namely in sorting mixtures of synthetic nanoparticles. Loading and unloading, while the rotor is spinning, avoids perturbations during acceleration and deceleration periods, and thus makes a vital contribution to sorting accuracy. Nowadays one can synthesize nanoscale particles in a wide variety of compositions and shapes. A prominent example for this are “colloidal molecules” or, generally speaking, defined assemblies of nanoparticles that can appear in varying aggregation numbers. Fractionation of such multimodal colloids plays an essential role with regard to their organization into hierarchical organized superstructures such as films, mesocrystals and metamaterials. Zonal rotor centrifugation was found to be a scalable method of getting “colloidal molecules” properly sorted. It allows access to pure fractions of particle monomers, dimers, and trimers, just as well as to fractions that are essentially rich in particle tetramers. Separations were evaluated by differential centrifugal sedimentation, which provides high-resolution size distributions of the collected nanoparticle fractions. The performance achieved in relation to resolution, zone widths, sorting efficiencies and recovery rates clearly demonstrate that zonal rotor centrifugation provides an excellent solution to the fractionation of nanoparticle mixtures.

## Abstract 2

Hollow bowl-shaped rotors allow for efficient fractionation of nanoparticle mixtures at large scale.

## Graphical abstract

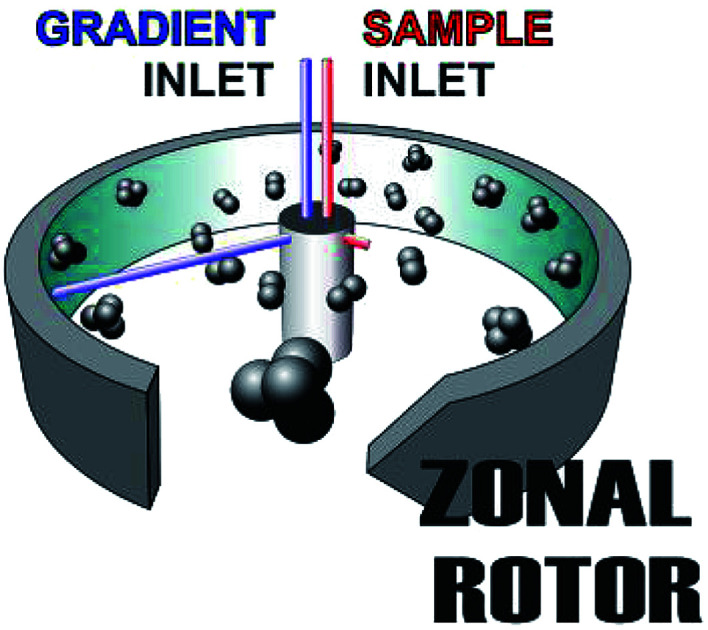

## Introduction

The enormous potential of nanoparticles relates to the extraordinary properties and functions of nanoscale objects, which open up a broad range of applications.^1^ Many of these properties are tied to size, shape and composition.^2^ Programmed assembly of nanoparticles into hierarchically organized superstructures, for example, receives growing attention as a bottom-up strategy to build functional materials. Consequently, various routes have been established to fabricate nanoparticle superstructures.^3^ However, the formation of well-ordered nanoparticle arrays remains a challenge because nanoparticles are rarely uniform. In general, they exhibit distributions in size, shape, and composition.^4^ All attributes and behaviors that depend on these three parameters vary among the particles as well. This includes their phase behavior, (catalytic) reactivity, optical, magnetic, or rheological properties, and last, but not least, nanotoxicity.^5^ Based on this, there is an urgent need for nanoparticles with narrow distributions in size, shape and composition. This might be achieved by precise adjusting of experimental parameters during synthesis. However, this is not always possible or polydispersity remains, albeit at a lower level. Post-synthesis fractionation of nanoparticles is thus highly appealing. Suitable methods that can produce narrowly-dispersed particle fractions include ultrafiltration,^6^ electrophoresis,^7^ field-flow fractionation,^8^ and size exclusion chromatography.^9^ These methods can be used for separations on a small scale.

The most common technique for isolating particles is that of differential centrifugation.^10^ In this method, a homogeneous suspension of the particles is centrifuged until the particles form a pellet at the bottom of the tube. Initially, the pellet primarily consists of the fastest sedimenting species but small particles near the bottom of the tube are collected as well. Given sufficient time, all particles will gradually end up in the pellet. This means that only a portion of the slowest sedimenting nanoparticles can be isolated in their pure state. Hence, differential centrifugation provides inherently poor resolution. Adequate sorting is only feasible if the sedimentation rates of the particles to be separated differ by at least a factor of 10.^11^

Consequently, there was a need for something better. Zonal centrifugation in a density gradient, originating from Brakke, is an elegant approach that became primarily popular in biological separations.^12^ This method eliminates the drawbacks of differential pelleting by keeping the particles always in the fluid phase and provides much higher resolutions. Two different types of zonal centrifugations were established:

(i) In rate-zonal centrifugation, the density of the gradient is at any position less than the buoyant density of the particles. The mixture of suspended particles to be separated is placed on top of the density gradient. Through centrifugation, the particle populations migrate as discrete zones because of differences between their sedimentation coefficients.^12^ The sedimentation coefficient of an individual particle is determined by its effective size, its buoyant density, and to a certain degree its shape. Rate-zonal centrifugation is highly efficient in sorting particles because particles whose sedimentation coefficients differ by as little as 15% can be separated.^13^

(ii) In isopycnic-zonal centrifugation, the density of the gradient exceeds that of the particles at one end. Sedimentation is continued until all particles reach a position where their buoyant densities equals the gradient density.^14^ In doing so, the particles are sorted entirely according to their densities. In the early days of the method, isopycnic-zonal centrifugation was applied to precisely determine the buoyant density of polystyrene latex particles by locating their zone within a known density gradient.^15^

The role of density gradients in rate-zonal separations is secondary, albeit indispensable. It is to eliminate currents (streaming) that have an adverse effect on banding particles into defined zones.^10^ The particles at the front of a zone encounter higher gravitational forces but also higher density and viscosity environments. Thus they may settle at the same rate as those in the trailing edge.^16^ Hence, the density gradient keeps similar particles together as migrating zones during centrifugation.

Zonal centrifugation requires rotors, in which the density gradient is always parallel to the force to which it is submitted.^17^ This can be ensured by using high-speed swinging-bucket rotors.^12^ Although rate-zonal density gradient centrifugation using swinging-bucket rotors is primarily used for biological separations, it has recently been recognized as a valuable tool for sorting nanomaterials as diverse as gold nanoparticles, magnetic nanocrystals, or chemically modified graphene.^18^

**Fig. 1:**
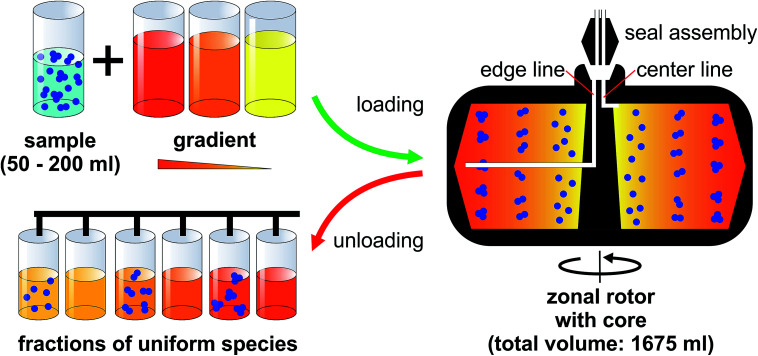
Zonal rotor centrifugation: a density gradient is introduced at the edge of a hollow rotor, while it is spinning at reduced speed. Loading starts with the lightest portion of the gradient first, followed by layers of increasing densities. Once the gradient fills the rotor completely, the sample suspension is introduced at the rotor core as the last material loaded. Separation is accomplished by sorting of the particles according to their sedimentation coefficients. At the end of the centrifuge run, the rotor speed is reduced again and its content is displaced out through the center exit by pumping a sufficiently dense solution into the edge line. Suspensions of sorted nanoparticles can be picked up using a fraction collector system.

Having access to a few milligrams of uniform nanoparticles through this technique is usually sufficient for analytical purposes. However, it is not enough for building materials from nanoparticles at a reasonable level. The solution to this is actually close at hand. The possibility to eliminate centrifuge tubes entirely by using hollow rotors was established by Anderson already in the 1960s.^19^ Several generations of rotors, referred to as zonal rotors, were developed within the Molecular Anatomy Program of the Oak Ridge National Laboratory.^17^ Most of the zonal rotors that were and are still commercially available are copies of the ones developed at Oak Ridge.^16,20^ Some of them allow for dynamic loading and unloading. This means that they can be loaded and unloaded while the centrifuge is running to avoid perturbations during acceleration and deceleration (Fig. 1).^16,17^ Zonal rotors were primarily developed and used for the separation of biological particles over a wide size range. They were successfully applied to the isolation of cells, organelles, viruses, ribosomes, serum proteins, and DNA.^11,14,21^ Fractionation using zonal rotors is thus capable to extend over several orders of magnitude in sedimentation coefficients (from 1 Sv to 100 000 Sv).^11,17^ The technique had found an important application in the commercial purification of influenza vaccines.^22^ Density gradient centrifugation using zonal rotors is nowadays largely superseded by chromatographic techniques in its original field.

It is quite astonishing that zonal rotors did not make the leap from biological separations into other fields. Zonal rotors have not been used for the fractionation of synthetic nanoparticles so far. To the best of our knowledge, the only ones who tested zonal rotors for the isolation of synthetic colloids were Wandrey and co-workers, EPFL Lausanne. In this context, it has to be mentioned that Nirschl and co-workers recently reported on a high-speed centrifugation technique based on magnetic bearing and drive technology.^23^ Their method, which is conceptually different from zonal rotor centrifugation, has also proven its potential in sorting synthetic nanoparticles.

Herein, rate-zonal density gradient ultracentrifugation in a zonal rotor is applied for the first time to the isolation of synthetic nanoparticles. To keep the designation of the method short, we will use the term “zonal rotor centrifugation” in the following.

The article is organized as follows: it highlights particularities of zonal rotor centrifugation. The main part demonstrates the capability of the method by a case study. Here we employ clusters of polymer nanoparticles as a model system to explore fractionation and isolation of particle mixtures with multimodal size distributions. Based on the universal nature of zonal rotor centrifugation, the results and insights attained are transferable to the separation of a broad range of nanoparticle systems.

## Experimental

### Materials

The colloidal clusters used throughout this work are identical to those in ref. 24. There, basic information on the preparation and morphologies of the clusters is to be found. d(+)-Sucrose (≥99, 5%) in a grade suitable for preparing optically clear density gradients was purchased from Carl Roth. Deionized water (resistivity > 18 MΩ) obtained from a reverse osmosis water purification system (Millipore Direct 8) was used throughout the entire studies.

### Methods

#### Zonal rotor centrifugation

An Optima XPN-90 Ultracentrifuge (Beckman Coulter) was adjusted by the manufacturer to enable mounting of a translucent protective shield in the rotor chamber, and leaving the top lid open while the centrifuge is running. The empty zonal rotor (Ti-15 Zonal Rotor with Standard Core, Titanium, 1675 ml, 32 000 rpm, 102 000*g*) was cooled to 20 °C, accelerated to 2000 rpm, and completely loaded at this speed. This is done by mounting a two-way fluid seal on the spinning rotor. The seal assembly enables dynamic loading by providing connections between rotor edge and core to tubings (Masterflex Silicone Tubing, size 16) leading outward from the rotor chamber. Step density gradients were prepared by pumping (Masterflex® L/S peristaltic pump; tubing sizes L/S® 16, Cole-Parmer) 10 sucrose solutions of increasing densities (F01: 155 ml each; F02: 152 ml each) at a flow rate of 40 ml min^−1^ to the rotor edge. Concentrations and densities of the individual gradient layers are gathered in Table S2.† After the sucrose gradient was in the rotor, an underlay (“cushion”) of concentrated sucrose solution (155 ml, 15 wt%) was pumped into the edge until the light end of the gradient began to flow out the core line, indicating that the rotor was completely loaded. The nanoparticle suspension (50 ml) to be separated was passed through 2.0 μm syringe filter (Membrex 25 PET, membraPure) and then pumped at 6.7 ml min^−1^ into the rotor core, forming there a sample zone centripetal to the gradient. Next, an overlay of water (50 ml) that displaces the sample from the core was pumped into the rotor. After removing the seal assembly and closing the rotor as well as the rotor chamber, the ultracentrifuge was accelerated to 32 000 rpm. After 45 min of centrifugation, the ultracentrifuge was decelerated back to 2000 rpm and the seal assembly was mounted. Unloading was accomplished through the core line by pumping a dense sucrose (20 wt%) at 40 ml min^−1^ towards the rotor edge. Fractions of 20 ml were collected using a Labocol Vario-4000 instrument (Labomatic Instruments).

#### Fraction analysis

The density of each fraction of nanoparticles dispersed in gradient material (sucrose solution) was measured at 20 °C using a DMA 5000 M density meter (Anton Paar). A photodiode array spectrophotometer (8453 UV-Visible Spectroscopy System, Agilent Technologies) was used to measure the absorbance of the nanoparticle fractions at a wavelength of 405 nm. All samples within a series were diluted with deionized water in a ratio of 1 to 3, provided that the absorbance of one of the samples was exceeding a value of 2. The sucrose did not influence the absorbance of the suspensions. Only after completion of the density and absorbance measurements, the sucrose was removed by exhaustive dialysis of the nanoparticle fractions against deionized water. In the latter case, this ensured that concentration changes as a result of dialysis can be ruled out.

The composition of all nanoparticle fractions was determined by differential centrifugal sedimentation (DCS). Measurements were carried out in an ultra-high resolution disk centrifuge (DC 24000 UHR, CPS Instruments). The instrument is equipped with a motor operating at 24 000 rpm and an optical detector calibrated at 405 nm. General information on DCS and its application in particle sizing is given in ref. 25. Specific information about the determination of sedimentation coefficients can be found in ref. 24. In the present case, absorbance of the nanoparticles during centrifugal separation of a given fraction was recorded. In doing so, different species within a fraction can be probed by their absorbance as a function of the particle settling time. The percentage composition of a given fraction is obtained *via* the peak areas assigned to the individual species.

Field emission scanning electron (FESEM) images were recorded on a Zeiss CrossBeam 1540XB microscope equipped with a field emission cathode operating at 3 kV. Specimen were prepared from dialyzed nanoparticle fractions by drying one drop of highly diluted suspensions on a clean silicon wafer at room temperature and coating with a platinum layer of 4 nm thickness using a sputter coater (Q150R ES Rotary-Pumped Sputter Coater, Quorum Technologies) to make the specimen conductive.

## Results and discussion

### Dynamic loading and unloading

**Fig. 2:**
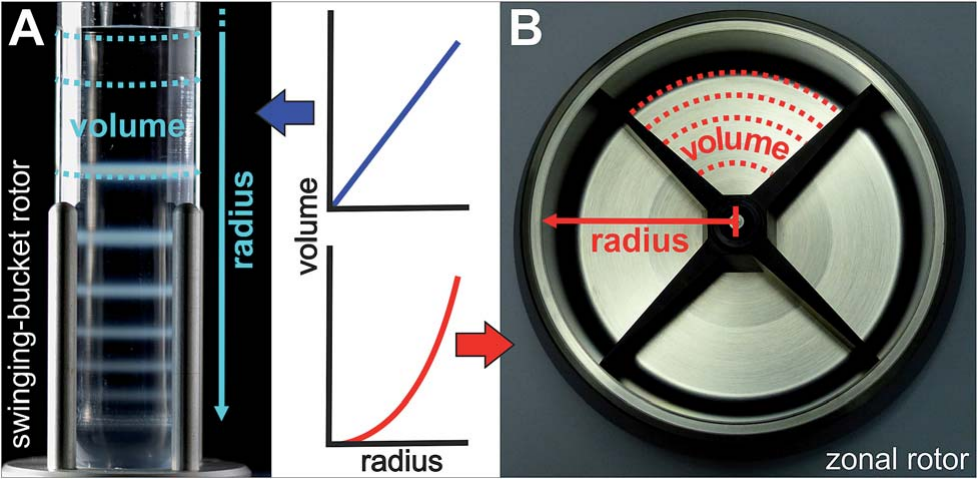
Comparison of rotors used for zone centrifugations. (A) In a swinging-bucket rotor, centrifugation tubes containing a density gradient are located in buckets, which can swing out and orient perpendicular to the axis of rotation. Different particle populations migrate as discrete zones through the gradient during centrifugation and will be separated from each other at this disposal. Although wall effects are drastically reduced as compared to fixed-angle rotors, only the particles far from the lateral wall will sediment directly to the bottom of the tube. (B) Wall effects are eliminated when using a zonal rotor, which can be regarded as a 360° extension of a swinging-bucket tube. Lateral walls are replaced by radial septa, which enables unconstrained sedimentation into sector-shaped compartments providing radial dilution. Rotor geometry has a considerable effect on the shape of a density gradient. A density gradient prepared linear with volume will assume a concave profile with radius when loaded into a zonal rotor, whereas it will be also linear with radius when placed into a centrifuge tube of a swinging-bucket rotor.

A zonal rotor may be thought as the derivative of a swinging-bucket tube by extending its lateral walls by 360° to form a cylinder (Fig. 2B).^11^ Replacing lateral walls minimizes undesirable wall effects on resolution. The bowl-shaped rotor contains a central core with typically four radial septa dividing the interior space into four sector-shaped compartments. The primary task for the core is providing channels that connect the edge and center of the rotor with the lines of the seal assembly. Furthermore, the division into compartments curbs swirls within the gradient, which is particularly important during acceleration and deceleration, and when loading and displacing the gradient. For these reasons, the rotor core is essential for enabling dynamic loading and unloading. It should be noted that also cores were designed with regard to gradient reorientation, and thus enabling loading and unloading at rest.^17^ However, zonal rotors that can be dynamically loaded have shown in practice to achieve particularly good separations.^16^

Precisely for this reason, the nanoparticle separations presented below were performed on a rotor/core setup that can be dynamically loaded and unloaded (Fig. 1). Sucrose solutions of various concentrations were employed as density gradient material because sucrose is uncharged, inert and does not absorb light at 405 nm. At first, the rotor is filled during rotation at reduced speed (2000 rpm). In doing so, the light end of a sucrose gradient is pumped through the seal assembly to the edge first, followed by layers of denser solutions that displace the lighter layers toward the core. When the gradient is fully loaded, its dense end is displaced from the rotor wall by pumping in a high-density sucrose solution. This dense and viscous underlay, which is referred to as cushion, gathers up sedimenting particles, preventing precipitation on the wall. Loading through the edge line is completed when gradient fluid begins to flow out the center line. At this point, the aqueous suspension of the particles to be separated can be introduced through the center line, with a portion of the cushion being displaced and flowing out the edge line. Finally, the particle zone is pushed further inside by pumping in water. This light overlay displaces the particles from the wall of the rotor core into a heavier gravitational field. After removing the seal assembly and closing the rotor chamber, the zonal rotor is accelerated to a speed allowing for efficient separation of the nanoparticles. The rotor is decelerated back to reduced speed before the particles to be isolated reach the cushion. The fluid load is removed from the zonal rotor by introducing a dense solution through the edge line. This solution must have a density greater than the cushion density. In the separations described below, the gradients containing particle populations banded into discrete zones were portioned in aliquots of 20 ml, thus allowing comprehensive analysis of the fractionation level achieved.

### The particle mixture to be separated

**Fig. 3:**
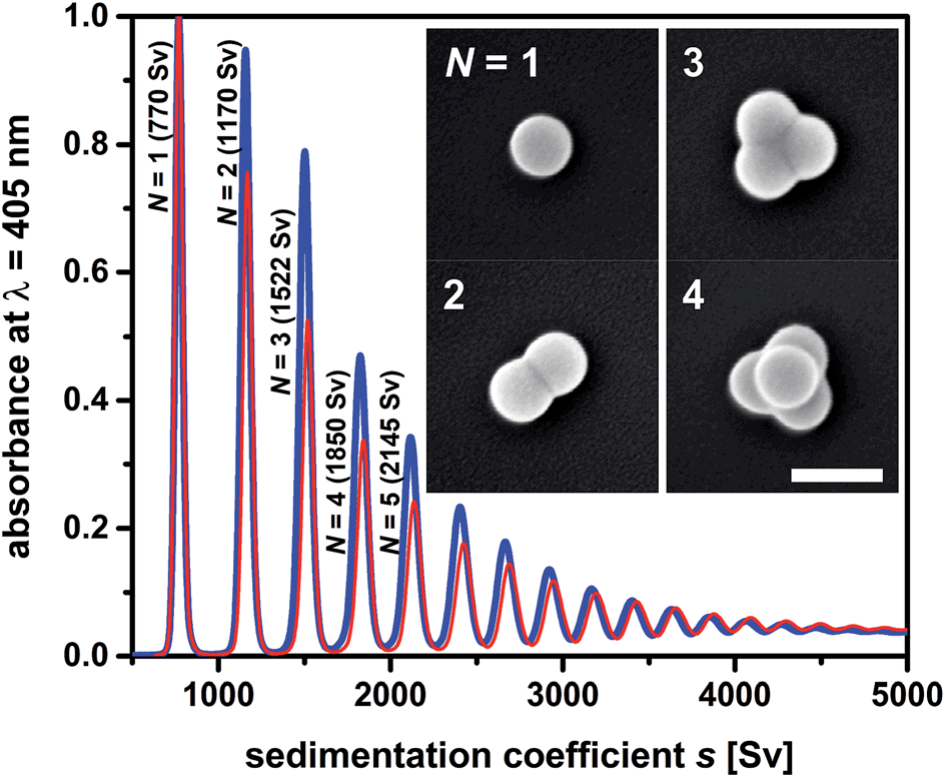
Sedimentation coefficient distributions as measured by differential centrifugal sedimentation (DCS). The two samples of “colloidal molecules” contain the same type of cluster species and differ only in terms of their numerical proportions. Mixture F02 (blue line) contains higher amounts of clusters with respect to particle monomers than mixture F01 (red line). Inset: FESEM micrographs of the particles to be isolated from the mixture (particle monomers *N* = 1, dimers *N* = 2, trimers *N* = 3 and tetramers with tetrahedral geometries *N* = 4). The scale bar represents 200 nm. More information on sample composition is found in Table S1.†

A straightforward strategy towards multimodal colloids is joining equal-sized spherical particles into stable clusters.^26^ Such clusters are often referred to as “colloidal molecules” because they may exhibit configurations similar those observed in true molecules.^27^ Their preparation can be based on the agglomeration of particles dispersed in an emulsion.^28^ Fixation of the particles to the droplet surfaces is ensured by the Pickering effect, which is driven by surface free energy. The particles trapped on each droplet pack in a stable cluster during gentle evaporation of the droplet phase. Using this approach, one can produce defined clusters with global dimensions in the colloidal regime. The statistical distribution of the number of particles on the droplets provides the framework needed to obtain supraparticles that differ in the number of constituents, and therefore also in size and shape (Fig. 3). The mixture of single particles, particle dimers, trimers, tetramers, and so forth presents an ideal testing system to probe fractionation of multimodal colloids using zonal rotor centrifugation. Recently, we reported on a modular approach that allows to precisely predict the sedimentation and diffusion coefficients of such particle clusters.^24^ In this approach, the clusters are treated as assemblies of overlapping shells of small friction elements. The latter is based on hydrodynamic bead-shell modeling established by Garcìa de la Torre.^29^ The sedimentation and diffusion coefficients predicted by this strategy agree very well with experimental data.^24,30^ As a result, knowledge about the hydrodynamic quantities can be used when defining experimental parameters for centrifugal separations.^31^ With increasing aggregation number, differences in sedimentation coefficients of the particle clusters become smaller (Fig. 3). Consequently, a multimodal mixture of “colloidal molecules” is highly suitable to explore the limits of centrifugal separation techniques.

Beyond fundamental insights in nanoparticle fractionation, sorting of “colloidal molecules” is an important task in itself. Currently, getting “colloidal molecules” properly separated presents a major bottleneck regarding their use as elementary units for hierarchically organized materials.^32^

Motivated by this, the potential of zonal rotor centrifugation for sorting “colloidal molecules” is explored. The “colloidal molecules” used for this purpose are assemblies of 144 nm sized spherical polymer latex particles. Assembly into clusters using the emulsion-assisted approach was performed along the lines given recently.^33^ Detailed information on the clusters, their morphologies, and their hydrodynamic behavior is found in ref. 24. It should be noted that the clusters are robust, once the disperse phase is completely removed. The assemblies do not disintegrate with time into their constituents, even when using ultrasound.^24^

### Capacities

In the following the fractionation of “colloidal molecules” is explored using a Beckman Coulter Ti-15 zonal rotor. Comparable separations carried out in a swinging-bucket centrifuge rotor are described in ref. 24 and 33. Admissible gradient volumes in swinging-bucket rotors do typically not exceed 30 ml, which limits sample volumes to 3 ml (Fig. 2A). The lower the difference between particle density and gradient density, the higher is the mass of particles that a zone can host.^17^ Given a 1 wt% suspension of polymer particles (density close to 1 g cm^−1^), the total amount of particles to be fractionated is about 30 mg. Although such quantities are adequate for many analytical purposes,^24,30^ the amount of “colloidal molecules” recovered in their pure form is by far not sufficient for building hierarchically organized materials. Moreover, the capacities are much smaller if particles with higher densities such as inorganic particles have to be separated.

Although not examined before, it is evident that the yield can be boosted by using zonal rotors. The capacity of the zonal rotor used here is 1675 ml, which permits sample volumes of 50 to 200 ml (Fig. 2B). Let us assume again a 1 wt% aqueous suspension of polymer particles. In this case between 500 and 2000 mg of “colloidal molecules” could be fractionated in a single run. This should allow making “colloidal molecules” available in quantities that are sufficient for building superstructures.^32^ It is clear that a suitable balance between resolution, on the one hand, and capacity, on the other hand, has to be found. Resolution is benefitting from having the particles in a narrow starting zone, whereas a broader zone can host more particles.^16^

In the following, separations of “colloidal molecules” are explored on the basis of two experiments, hereinafter referred to as fractionations F01 and F02. A total of 90.0 mg of “colloidal molecules” dispersed in 50 ml of deionized water were fractionated in experiment F01. The potential for higher capacities was explored in experiment F02 by sorting 621.6 mg of “colloidal molecules” (also suspended in 50 ml of water). Table S1† provides compositions of the two colloidal mixtures utilized in each case, expressed as the number and mass percentages of the individual components. The amount of a given particle cluster decreases with rising number of constituent particles *N*. The objective was the separation of the particle monomers, dimers, trimers, and tetramers from larger species and the recovery of the four particle populations in as pure form as possible.

### Density gradient and its profile

It is known from diverse biological separations that the shape of the density gradient can have a great influence on the separation in terms of resolution and capacity.^16,34,35^ Gradients that are linear with volume are most commonly used in density gradient centrifugations. In this context it must be kept in mind that within a zonal rotor equal volumes will form layers of decreasing thickness with increasing distance from the rotor center. In other words, a gradient that is linear with volume is concave with rotor radius (Fig. 2). This difference, which does not exist with swinging-bucket rotors, has to be considered when working with zonal rotors. The density gradients were prepared by layering sucrose solutions of equal volume. In experiment F01, the sucrose concentration was raised incrementally from 7 wt% to 12 wt%. Measurements of the density of the fractions collected after unloading of the zonal rotor, clearly showed that diffusion of the solute had smoothed out the density steps. Thus, the gradient used in experiment F01 can be regarded as linear with volume (Fig. 5A) and as concave with radius (Fig. 5B).

General statements on the optimum profile of the density gradient are difficult to make because it depends upon the particle populations to be separated. In the case of mixtures of different RNA species, better separations were achieved in sucrose gradients with steeper density profiles.^36^ In separations that focus on the isolation of a specific particle population, it is assumed that gradients that are convex with radius should give best results.^16,37^ The steepest part of the gradient is located next to the starting sample. For the reasons indicated above, a specific particle population is kept closely together if the density and viscosity environment experienced by the leading and the trailing edge of a zone are rather different. Convex gradients thus provide narrow zone widths and highest capacity at the outset of the sedimentation path. During their way down the sedimentation path the zones will broaden and radial dilution in a zonal rotor will decrease the concentration within a zone (Fig. 2). In this regard, capacity is in inverse relationship to the spatial expansion of a distinct zone.^16,37^

To further investigate if a convex gradient might be also advantageous for the separation of “colloidal molecules”, a step gradient that evolved with time into a convex gradient (both with radius and volume) was chosen in experiment F02 (Fig. 5C and D).

### Centrifugation times

An iterative method can be used to predict the optimum time of centrifugation because the sedimentation coefficients of the “colloidal molecules” and the density gradient profile are precisely known (Fig. 3).^31^ Routines worked out for the analysis of sedimentation velocities such as SEDFIT can be used as well.^38^ One shortcoming is however the need to take acceleration and deceleration periods into account when calculating effective times of centrifugation (Fig. S1†). The latter can be done by integrating the square of revolutions per minute (rpm^2^) over time and division by the square of the rpm value at maximum speed (32 000 rpm).^36^ The resulting equivalent times of top speed operation were 39.2 min in experiment F01, and 38.6 min in experiment F02. These centrifugation times have proven as an optimum solution for sorting particle monomers, dimers, trimers, and tetramers from larger *N*-mers (Fig. 5).

### Absorbance profiles and zone widths

**Fig. 4:**
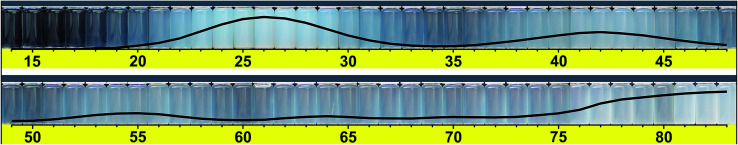
Photographs of fractions 14 to 83 (lined up in two rows) recovered from the zonal rotor in experiment F01. Fractions 1 to 13 are not shown because they do not contain any particles. The black line reflects the absorbance of the nanoparticle fractions at a wavelength of 405 nm. The much higher amount of particles in experiment F02 did not allow for a comparable assessment of the separation by visual inspection.

**Fig. 5:**
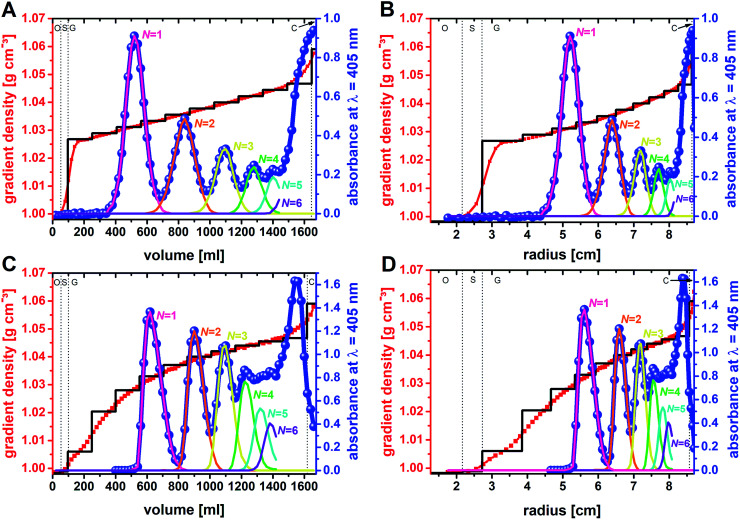
Fractionation of multimodal mixtures of “colloidal clusters”: gradients consisting of multiple density steps (black lines) were loaded into the zonal rotor. With time, the step gradient changed into a continuous profile (red lines and squares), as evidenced by density measurements of the fractions extracted from the rotor. The blue dots represent the absorbance of the fractions. The absorbance profiles (blue lines) thus reflect the separation of the various particle populations, which differ in the number of constituents *N*. Centrifugation times were optimized for the isolation of clusters with *N* ≤ 5. In doing so, larger species are accumulated near the rotor edge. Experiment F01: separation of 90 mg of “colloidal molecules” using a density gradient that is linear with volume (A), and concave with radius (B). Experiment F02: separation of 621.6 mg of “colloidal molecules” using a gradient that is convex both with volume and radius (C and D). See text for further explanations. Compositional analysis of each individual fraction by DCS gave the decomposition of the absorbance profiles into discrete contributions. The letters O, S, G, and C refer to the subdivision of the fluid load of the zonal rotor into overlay (O), sample zone (S), density gradient (G), and cushion (C). See experimental section for further explanations.

**Fig. 6:**
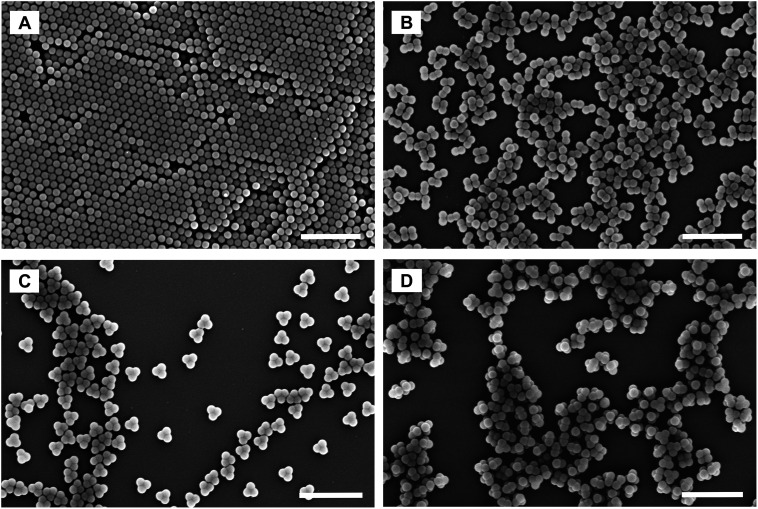
FESEM micrographs of distinct fractions collected from the zonal rotor. (A–C) represent fractions that exclusively consist either of particle monomers, dimers, or trimers. (D) refers to a fraction that is essentially rich in particle tetramers, but also contain minor portions of trimers and pentamers. The selected fractions correspond to local maxima of the absorbance profiles shown in Fig. 5. Scale bars represent 1000 nm.

Unloading of the zonal rotor was achieved by displacing the particle populations banded into discrete zones through a dense sucrose solution. In order to allow for an in-depth analysis, 83 fractions, 20 ml each, were collected (Fig. 4). Measurements of the density and the absorbance at 405 nm gave the profiles shown in Fig. 5. The absorbance profiles reflect the separation of the “colloidal molecules” into monomers, dimers, trimers, tetramers, and pentamers. This is also confirmed by FESEM analysis of the fractions that can be assigned to the local absorbance maxima (Fig. 6 and S4†).

In order to maximize the sedimentation path (here: *r*_max_ = 87.9 mm; Beckman Coulter Ti-15 zonal rotor with standard core) for the mentioned species, it was consciously accepted that *N*-mers with larger aggregation numbers will accumulate near the rotor edge (adjacent to the cushion of high sucrose density). The shapes of the zones of banded particle populations differ between the two separations (Fig. 5). When plotting absorbance against volume, the bands in experiment F01 correspond to Gaussian distributions, whereas those in experiment F02 have asymmetric shapes. The different band shapes reflect the different profiles of the density gradient. The profile, which is linear with volume in experiment F01, did not distort the normal distribution of the particles. If, however, a gradient convex with volume is applied as in experiment F02, particles in the trailing edge of a zone face a steeper part of the gradient than those at the leading end. The latter results in asymmetric concentration profiles.

The sample had an initial zone width of 5.5 mm when placed into the rotor. Zone broadening is likely to occur because of Brownian diffusion of the particles.^24,30^ However, the centrifugal field in combination with the density gradient limits zone broadening by diffusion. The absorbance profiles of the fractions collected from the rotor provide insights into the final zone widths when the separation is completed. The band widths can be related to twice the standard deviation of the band assigned to a distinct particle population. With regard to experiment F01, the band widths expressed as twofold standard deviation are 4.8 mm for particle monomers, 4.4 mm for dimers, 3.4 mm for trimers, 2.6 mm for tetramers, and 2.0 mm for pentamers. This can be explained by a significant band narrowing when the particles enter into the gradient. Because of the steep increase in the density profile (Fig. 5A) at the boundary of sample area and gradient, the particles met an environment of higher density and viscosity resulting in an immediate narrowing of the migrating zones once they enter into the gradient. Additional zone narrowing is to be expected during the migration of the zones up the concave density gradient. Apart from the shape of the density gradient, zone width is also affected by Brownian diffusion of the nanoparticles. This is reflected in broader zone widths of clusters with decreasing number of constituents. The latter have higher diffusion coefficients and occur in larger concentrations (Fig. 3 and Table S1†).

Similar observations can be made in experiment F02. Here zone widths are 4.1 mm for monomers, 3.1 for dimers, 2.8 for trimers, 2.5 mm for tetramers, and again 2.5 for pentamers. It is evident that the higher total amount of particles to be separated had no adverse effect on zone width. In line with the above discussion, the convex shape of the gradient (both in volume and radius) led to smaller zone widths for particle populations that are extracted from steeper parts of the gradient. This, however, reduces sorting efficiency of populations such as tetramers that are collected from outer parts of the zonal rotor where the density profile is rather flat. Thus, the comparison between the two fractionations shows very clearly that separation of colloidal mixtures can benefit greatly from custom-tailored density gradient profiles.

### Resolution

Resolutions achieved in density gradient centrifugations were discussed in various biological separations. The power of resolving two adjacent zones is typically defined as the radial distance of the zone centers divided by the sum of the standard deviations of the two radial particle distributions involved.^13,39,40^ In the particular case of a zonal rotor, radial distances should be replaced by gradient volumes.^35^ The resolution achieved is then defined as:1
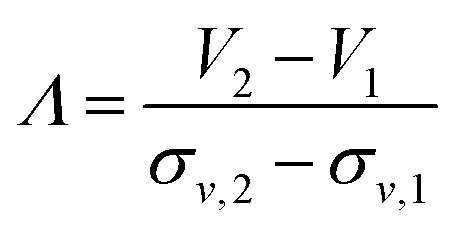
where *V*_2_ − *V*_1_ is the volume separating the zone centers and *σ*_*v*,1_*σ*_*v*,2_ are the standard deviations of the particle distributions with respect to volume. These quantities can be extracted from the absorbance profiles depicted in Fig. 5.‡‡Asymmetry in band shapes were considered when calculating resolutions. In that case, solely the halves of the particle distributions that can potentially overlap were taken into account when calculating standard deviations.

In experiment F01 based on a linear density gradient, resolutions achieved are 2.5 for monomers and dimers, 2.1 for dimers and trimers, 1.7 for trimers and tetramers, and 1.4 for tetramers and pentamers. The achieved separations are thus clearly above the resolution limit of 1, where the sum of the standard deviations of the particle distributions is identical to the volume between two zones (eqn (1)). As expected, resolution gets smaller with decreasing differences in sedimentation coefficients of the species to be separated (Fig. 3).^24^ Best resolution is thus achieved for separation of monomers from dimers. In this specific case, an even better resolution of 2.6 was achieved using a convex gradient (experiment F02). This is however at the expense of the other populations. Resolution achieved in sorting dimers from trimers was 1.9 and thus slightly below the resolution achieved with the linear gradient. This is also reflected by the resolution of particle trimers and tetramers (1.5 *vs.* 1.7). Resolution has reached its specified limit (1.0) with separating tetramers and pentamers when using the convex gradient.

### Analysis of cluster fractions

**Fig. 7:**
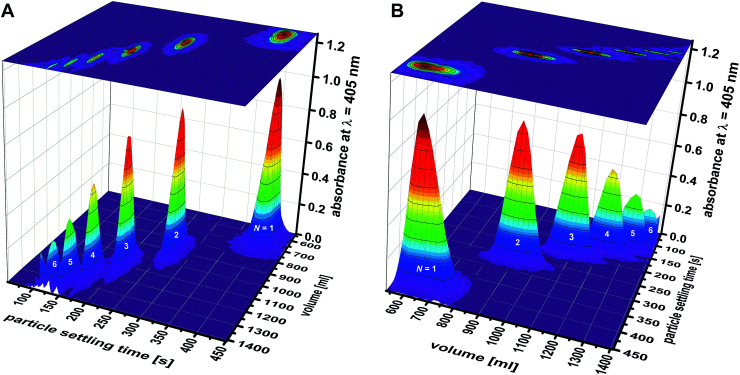
Separation of mixtures of “colloidal molecules”: absorbance *versus* particle settling time was measured by DCS for each fraction collected in experiment F02. The individual graphs depicting particle distributions were pooled into a 3D graph, which is shown from two different perspectives (A and B). The graph shows the efficiency that is achieved in sorting clusters of up to six constituent particles (*N* = 1: monomers; *N* = 2: dimers; *N* = 3: trimers; *N* = 4: tetramers; *N* = 5: pentamers; and *N* = 6: hexamers). Comparable 3D graphs related to experiment F01 are found in the ESI (Fig. S4†).

In addition to the total absorbance determined by a spectrophotometer, the fractions were analyzed by differential centrifugal sedimentation (DCS). This analytic fractionation method is complementary to analytical ultracentrifugation. It can be used to determine particle size distributions.^25^ Here, DCS was used for a compositional analysis of the initial samples (data gathered in Table S1†). DCS analysis also gave precise knowledge about the composition of the 83 fractions recovered from the zonal rotor (Fig. 7). The primary data acquired in DCS is the absorbance (at 405 nm) as a function of the sedimentation time. Different particle populations can be resolved as discrete bands.^24^ Integration of absorbance over sedimentation time thus gave the percentage of a distinct particle population to the total absorbance caused by all species of a distinct fraction. The deconvolution of the absorbance profile of the mixtures of “colloidal molecules” into contributions of distinct species is a clear proof of the high accuracy achieved (Fig. 5). For each fraction, the absorbance assigned to a specific type of particles can be set in relation to all fractions that contain this species. The percentage obtained this way can be directly converted into the number and mass of particles because their total amount across all fractions is known. As a result, the number and mass distributions of the different populations of “colloidal molecules” across the 83 fractions recovered from the zonal rotor can be precisely assessed (Tables S3 and S4†). This, in turn, is essential with regard to calculating recovery rates and varietal purities (see below).

The absorbance profiles measured by DCS for each of the 83 fractions can be pooled into a 3D graph, which gives a visual impression of the separation achieved (Fig. 7). Qualitative differences among experiments F01 and F02 can be seen as well. At this stage, however, preference should be given to a quantitative evaluation of the sorting efficiencies achieved.

### Recovery rates and varietal purities

Table 1 expresses the quality of the separations in numbers. Fractions rich in a distinct particle population can be pooled in order to maximize the amount of recovered particles. Recovery rates are defined as percentages of the number of a certain species recovered with respect to the total number of this population that was originally present in the mixture to be separated. In addition, varietal purities can be calculated as the percentage of the distinct particle species in the total number of particles found in the merged fractions. Relating recovery rates to varietal purities provides an effective indicator for the quality of the separation achieved. In both separations, it is found that more than half of the monomer, dimer, and trimer particles can be recovered at 100% purity (Fig. 6). Moreover, a vast majority of monomer, dimer and trimer particles can be recovered during the separation, provided that 95% varietal purity is sufficient for the intended purpose. The recovery of tetramers in pure form is more challenging. The sedimentation coefficient of a tetramer deviates just by 18% from the one of a trimer, and by 16% from the one of a pentamer.^24^ In the literature the limit specified for zonal separations is seen in the range of 15% to 30%, depending on the size of the species to be separated.^13,39^ It is therefore necessary to work out the physical limits of the method to optimize separations of tetramers. Nevertheless, the examples shown here demonstrate that it is possible to get fractions that are essentially rich in tetramers (Fig. 6D and S4†). This is remarkable especially considering the fact that further improvements can be expected from systematic optimization of the gradient profile. Separations just recently achieved in swinging-bucket rotors can be taken as a clear indication that the limits of what is possible are not yet reached.^24^

**Table 1:** Percentage recovery rates (RR) of *N*-membered particle clusters at various degrees of purity (*P*)

	*P* = 100%	*P* > 99%	*P* > 95%	*P* > 90%	*P* > 80%
**F01** a					
*N* = 1	RR = 86%	100%	100%	100%	100%
*N* = 2	58%	88%	98%	99%	100%
*N* = 3	50%	75%	90%	96%	99%
*N* = 4	0%	0%	44%	67%	79%

**F02** b					
*N* = 1	RR = 80%	100%	100%	100%	100%
*N* = 2	70%	96%	99%	100%	100%
*N* = 3	49%	62%	88%	92%	98%
*N* = 4	0%	0%	0%	0%	63%

a 90.0 mg of particle clusters separated in a linear (in volume) density gradient.

b 621.6 mg of particle clusters separated in a convex (in volume) density gradient.

The data presented in Table 1 shows that improved separation efficiencies can be achieved for dimer particles with convex sucrose gradients, whereas gradients, which are linear with volume (concave with radius) are better suited for tetramer particles (Fig. 7). This can be explained by the above statements on the density gradient profiles used in experiment F01 and F02. Using convex gradients results in asymmetric concentration profiles of the particle zones, which is reflected in the shapes of the absorbance bands shown in Fig. 5C. The latter is less critical for particle dimers whose sedimentation coefficients differ from trimers by 30%. Consequently, the separation of the dimer particles gains from the higher capacity and the resolving power of a convex gradient. In the case of the tetramers, the symmetric (Gaussian) shape provided by linear gradients reduces the overlap between the trimer and tetramer bands (Fig. 5A). Separations of particle mixtures with small differences in their sedimentation coefficients thus seem to benefit from linear gradients, which provide constant capacity and resolution over the entire sedimentation path.

## Conclusions

Zonal rotor centrifugation constitutes a powerful tool, which gives access to uniform nanoparticles to be used both in fundamental science and for practical applications. In case of “colloidal molecules”, the method permits efficient sorting of monomer, dimer and trimer particles from one another – even at 100% varietal purity. Limitations are just reached if differences in sedimentation coefficients among two particle populations are small (less than 20%). Given further optimization of density gradient profiles, it should nevertheless be entirely conceivable to drastically improve varietal purity of such species as well, or at least get them separated from single particle and small clusters.

Regarding future perspectives, further efforts should be geared to predict run parameters and density gradient profiles that offer an ideal compromise of sorting efficiency and sorting capacity. Again, “colloidal molecules” may serve here as a “gold standard” for nanoparticle mixtures because, based on their defined geometries, hydrodynamic properties of “colloidal molecules” can be precisely assessed – both by experiment and hydrodynamic calculations. Considering the extension to inorganic particles, having suitable gradient materials is of substantial importance. This is due to the higher density of inorganic particles as compared to polymer colloids. Among the materials applied for preparing density gradients, aqueous solutions of cesium chloride are the one providing the highest densities of up to 1.91 g cm^−1^. Apart of the fact that using a salt has detrimental effects on colloidal stability, the density of the gradient material is much lower than those of gold nanoparticles (19.32 g cm^−1^, if organic stabilizers are disregarded). Fortunately, recent experiments using swinging-bucket rotors showed that even gold nanoparticles can be properly sorted in glycerol/water density gradients.^41^ Here viscosity does what density cannot do alone. It slows down sedimentation and diffusion and makes sorting of heavy particles in centrifugal fields more efficient. This ultimately opens the way in using zonal rotor centrifugation for a broad range of organic and inorganic particles, which vary considerably in their buoyant densities.

Beyond rate-zonal separations, isopycnic separations can be also performed in zonal rotors. This opens up perspectives for sorting mixtures of particles with different densities at a large scale. On the other hand, the method may also prove as an efficient tool for the fractionation of hybrid particles, such as core–shell or composite particles.

Despite the progress already achieved, there is thus plenty of room left for further optimizations and extension of the method to applications beyond the separation of “colloidal molecules”. In particular, major improvements in terms of resolution and capacity can be expected from targeted optimization of the density and viscosity profile of the gradient. Due to the widespread use of nanoscale particles, a vast number of practical applications would substantially benefit from zonal rotor centrifugation making nanoparticles available at superior quality.

## Conflicts of interest

There are no conflicts to declare.

## Supplementary Material

RA-009-C9RA05140F-s001
