# Supplementary material for: Zonal rotor centrifugation revisited: new horizons in sorting nanoparticles
Source: RSC Adv. 2019 Sep 2;9(47):27549–59. doi: 10.1039/c9ra05140f (PMC9070787; doi:10.1039/c9ra05140f)
Supplement: RA-009-C9RA05140F-s001 [file RA-009-C9RA05140F-s001.pdf]

Electronic Supplementary Material (ESI) for RSC Advances.
This journal is © The Royal Society of Chemistry 2019

Supplementary Information

Zonal Rotor Centrifugation Revisited: New Horizons in Sorting Nanoparticles

Claudia Simone Plüisch,[a] Brigitte Bössenecker,[b] Lukas Dobler[a] and Alexander Wittemann*[a]

[a] *Colloid Chemistry, Department of Chemistry, University of Konstanz, Universitaetsstrasse 10, D-78464 Konstanz, Germany.*
E-Mail: alexander.wittemann@uni-konstanz.de
Web: https://www.chemie.uni-konstanz.de/wittemann/

[b] *Particle Analysis Center, Department of Chemistry, University of Konstanz, Universitaetsstrasse 10, D-78464 Konstanz, Germany.*

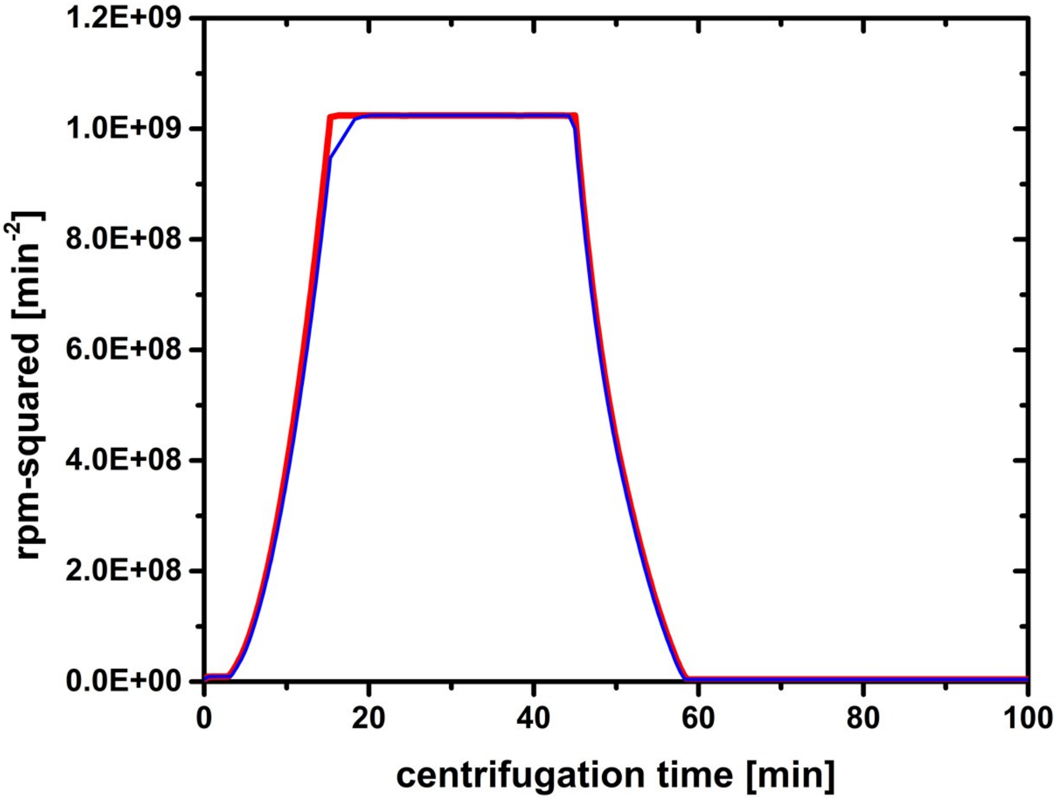

Fig. S1. Estimation of the effective time of centrifugation. Revolutions per minute-squared are plotted against centrifugation time (red line: F01 experiment; blue line: F02 experiment). Integration over time (within the time span between loading and unloading) and division by maximum revolutions per minute-squared gives the equivalent time to centrifugation at maximum speed, which is regarded as the effective time of centrifugation. See reference 25 in the main article for further explanations.

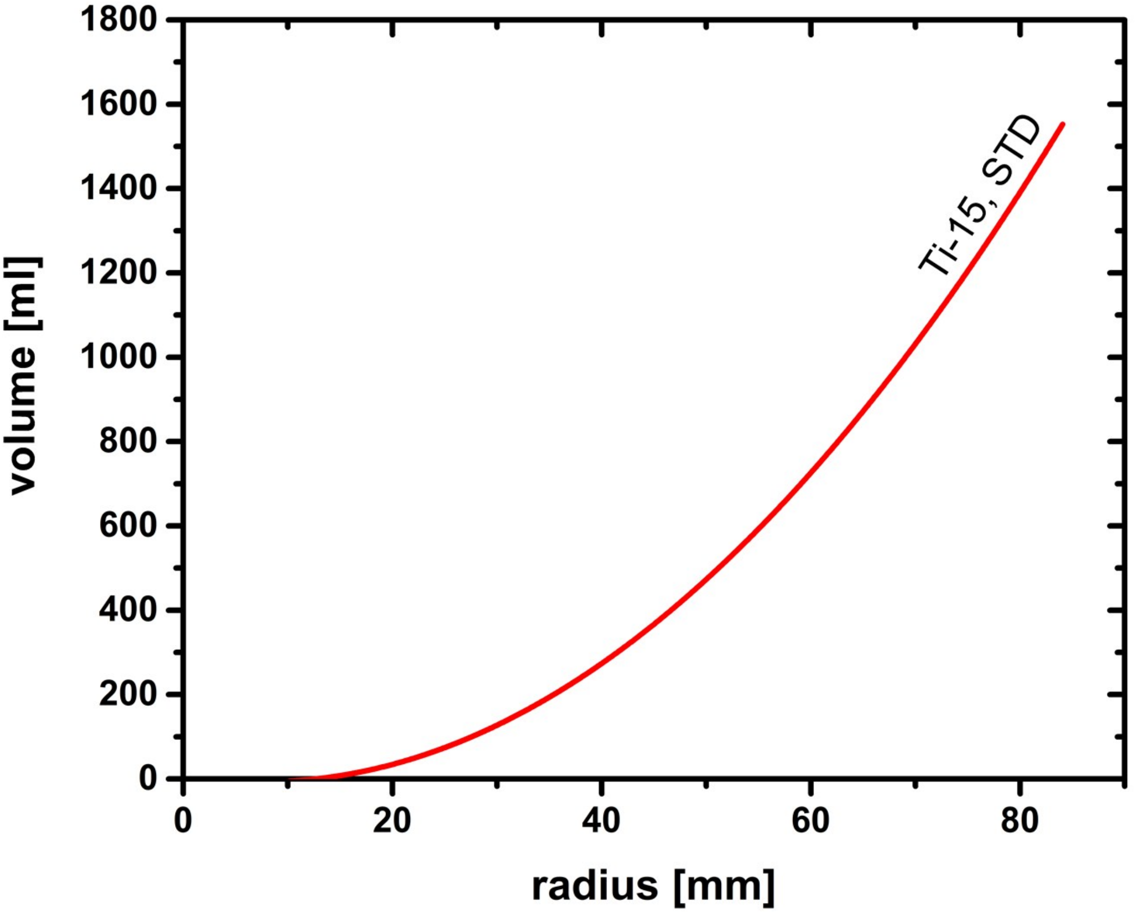

Fig. S2. Rotor and core geometry are factors that influence the volume-to-radius relationship of a zonal rotor. A graph showing exactly the volume-to-radius relationship for the rotor setup in this study (Beckman Coulter Ti-15 Zonal Rotor with Standard Core) can be found in the instruction manual (Beckman Coulter, *Zonal Rotors for Preparative Ultracentrifuges*, Fullerton, CA, 2007). The underlying (radius, volume) data of the scanned printed graph was extracted and fitted using a quadratic function, which reads as follows: $volume = 26.61 \cdot (radius - 1.27\ \mathrm{cm})^2 + 27.58 \cdot (radius - 1.27\ \mathrm{cm})$. This equation was used for any volume-to-radius conversions throughout the studies.

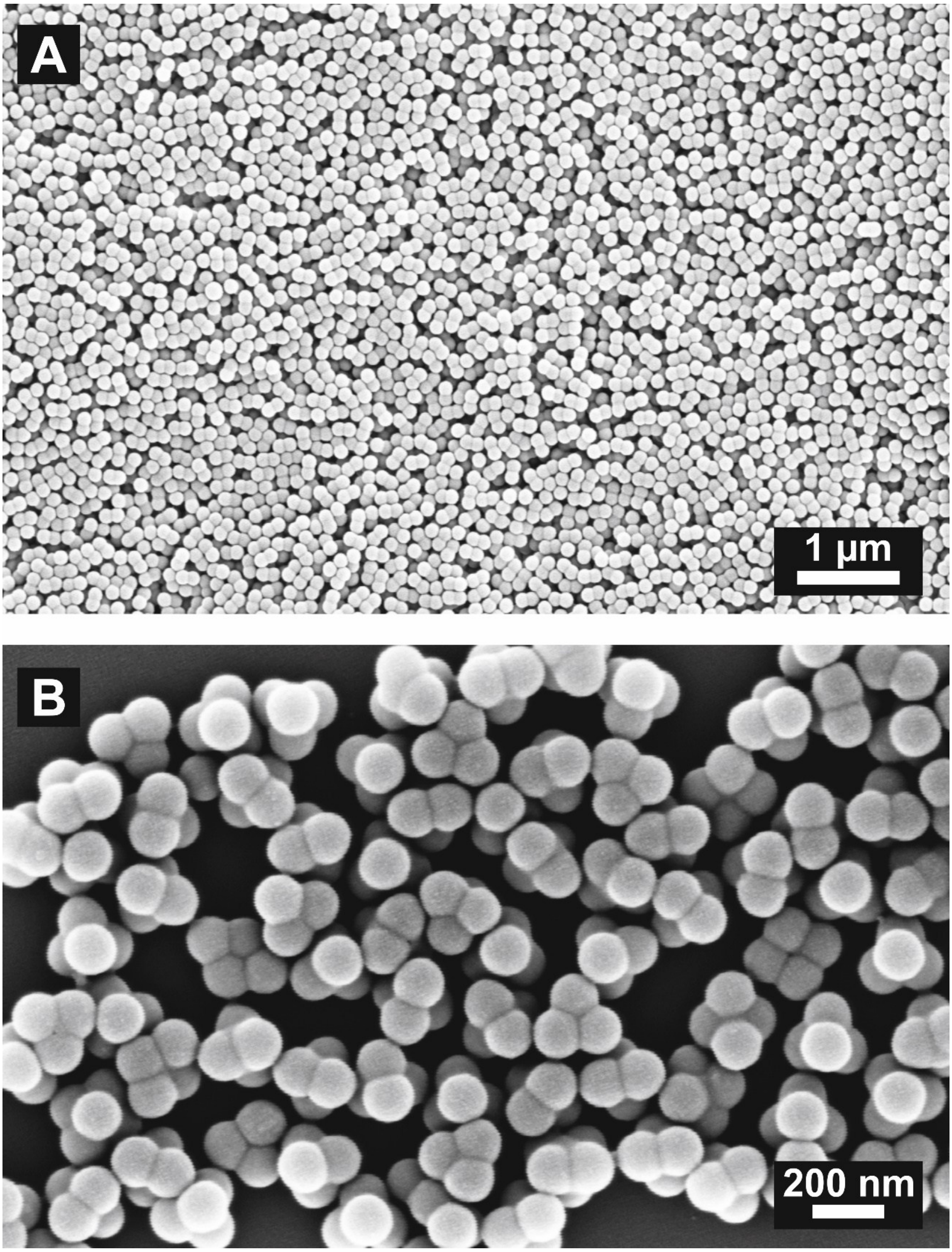

Fig. S3. FESEM micrographs complementary to those shown in the main manuscript. A) Fraction of pure particle dimers (“colloidal oxygen molecules”). B) A fraction that is essentially rich in particle tetramers (“colloidal P_4 molecules”). Both fractions were collected from experiment F01.

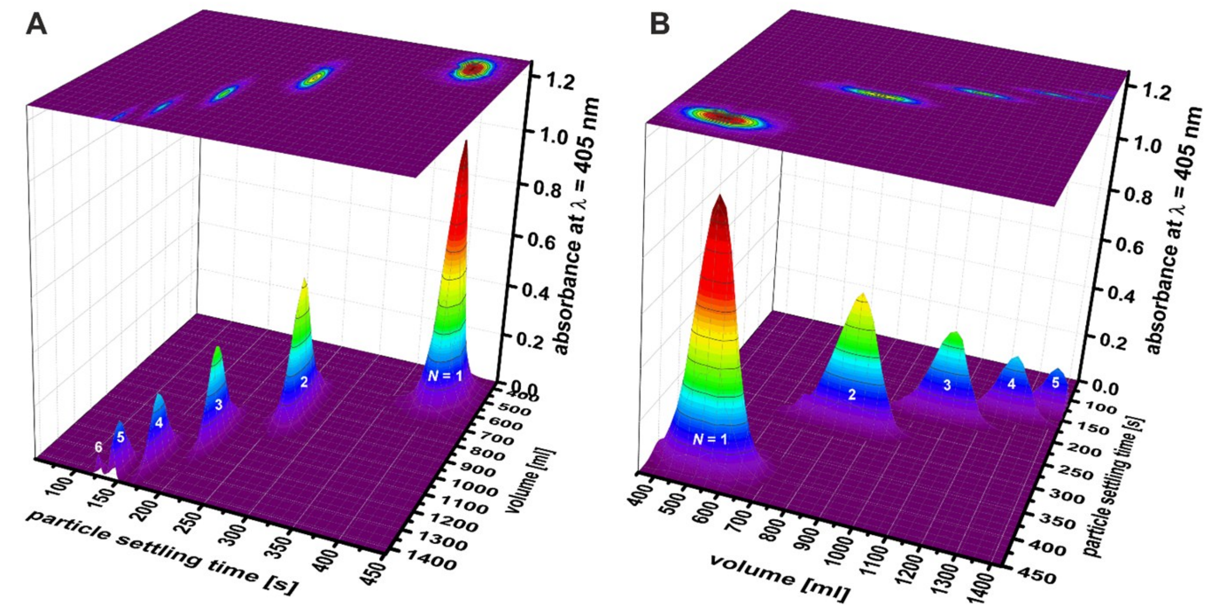

Fig. S4. Separation of mixtures of "colloidal molecules": Absorbance *versus* particle settling time was measured by DCS for each fraction collected in experiment F01. The individual graphs depicting particle distributions were pooled into a 3D graph, which is shown from two different perspectives (A, B). The graph shows the efficiency that is achieved in sorting clusters of up to six constituent particles (*N*=1: monomers; *N*=2: dimers; *N*=3: trimers; *N*=4: tetramers; *N*=5: pentamers; and *N*= 6: hexamers).

Table S1. Exact formulations of the mixtures of *N*-mers to be separated by zonal rotor centrifugation. The figures were determined by integration of particle size distributions measured by DCS.

F01[a)]	number fraction	weight fraction	F02[b)]	number fraction	weight fraction
N = 1	68.6%	40.7%	*N* = 1	59.4%	33.6%
N = 2	17.8%	19.7%	*N* = 2	21.3%	22.0%
N = 3	6.5%	10.6%	*N* = 3	10.0%	15.1%
N = 4	2.7%	6.0%	*N* = 4	3.8%	7.6%
N = 5	1.4%	3.7%	*N* = 5	2.0%	5.1%
N ≥ 6	3.0%	19.3%	*N* ≥ 6	3.5%	16.6%

[a)] 90.0 mg of *N*-mers in total [b)] 621.6 mg of *N*-mers in total

Table S2. Concentrations and densities (at 20°C) of the sucrose layers used in experiment F01 and experiment F02 for building the density gradients.

F01	sucrose conc. [wt%]	density [gcm^{-3}]	F02	sucrose conc. [wt%]	density [gcm^{-3}]
overlay	0	0.998	overlay	0	0.998
sample	0	0.999[a)]	sample	0	0.999[a)]
grad. layer 1	7.00	1.027	grad. layer 1	1.59	1.006
grad. layer 2	7.56	1.029	grad. layer 2	5.37	1.020
grad. layer 3	8.11	1.031	grad. layer 3	7.32	1.028
grad. layer 4	8.67	1.033	grad. layer 4	8.59	1.033
grad. layer 5	9.22	1.036	grad. layer 5	9.60	1.037
grad. layer 6	9.78	1.038	grad. layer 6	10.35	1.040
grad. layer 7	10.33	1.040	grad. layer 7	10.85	1.042
grad. layer 8	10.89	1.042	grad. layer 8	11.34	1.044
grad. layer 9	11.44	1.044	grad. layer 9	11.71	1.046
grad. layer 10	12.00	1.047	grad. layer 10	12.00	1.047
cushion	15.00	1.059	cushion	15.00	1.059

[a)] measured at 20°C using a DMA 5000 M density meter (Anton Paar)

Table S3. Mass composition of the fractions collected in experiment F01

Fraction N°	Volume [ml]	Monomers [mg]	Dimers [mg]	Trimers [mg]	Tetramers [mg]	Pentamers [mg]
18	360	**0.15**	0.00	0.00	0.00	0.00
19	380	**0.23**	0.00	0.00	0.00	0.00
20	400	**0.56**	0.00	0.00	0.00	0.00
21	420	**0.98**	0.00	0.00	0.00	0.00
22	440	**1.68**	0.00	0.00	0.00	0.00
23	460	**2.74**	0.00	0.00	0.00	0.00
24	480	**3.80**	0.00	0.00	0.00	0.00
25	500	**4.62**	0.00	0.00	0.00	0.00
26	520	**4.94**	0.00	0.00	0.00	0.00
27	540	**4.71**	0.00	0.00	0.00	0.00
28	560	**4.04**	0.00	0.00	0.00	0.00
29	580	**3.08**	0.00	0.00	0.00	0.00
30	600	**2.10**	**0.01**	0.00	0.00	0.00
31	620	**1.34**	**0.02**	0.00	0.00	0.00
32	640	**0.80**	**0.05**	0.00	0.00	0.00
33	660	**0.46**	**0.09**	0.00	0.00	0.00
34	680	**0.24**	**0.18**	0.00	0.00	0.00
35	700	**0.09**	**0.32**	0.00	0.00	0.00

36	720	**0.04**	**0.48**	0.00	0.00	0.00
37	740	**0.02**	**0.77**	0.00	0.00	0.00
38	760	0.00	**1.06**	0.00	0.00	0.00
39	780	0.00	**1.40**	0.00	0.00	0.00
40	800	0.00	**1.73**	0.00	0.00	0.00
41	820	0.00	**2.02**	0.00	0.00	0.00
42	840	0.00	**2.14**	0.00	0.00	0.00
43	860	0.00	**1.98**	0.00	0.00	0.00
44	880	0.00	**1.73**	**0.01**	0.00	0.00
45	900	0.00	**1.36**	**0.02**	0.00	0.00
46	920	0.00	**0.99**	**0.04**	0.00	0.00
47	940	0.00	**0.66**	**0.06**	0.00	0.00
48	960	0.00	**0.39**	**0.13**	0.00	0.00
49	980	0.00	**0.22**	**0.26**	0.00	0.00
50	1000	0.00	**0.08**	**0.39**	0.00	0.00
51	1020	0.00	**0.03**	**0.59**	0.00	0.00
52	1040	0.00	**0.01**	**0.84**	0.00	0.00
53	1060	0.00	0.00	**1.09**	0.00	0.00
54	1080	0.00	0.00	**1.25**	0.00	0.00
55	1100	0.00	0.00	**1.28**	0.00	0.00
56	1120	0.00	0.00	**1.18**	0.00	0.00
57	1140	0.00	0.00	**0.89**	**0.03**	0.00
58	1160	0.00	0.00	**0.63**	**0.06**	0.00
59	1180	0.00	0.00	**0.43**	**0.12**	0.00
60	1200	0.00	0.00	**0.24**	**0.26**	0.00
61	1220	0.00	0.00	**0.13**	**0.41**	0.00
62	1240	0.00	0.00	**0.06**	**0.64**	0.00
63	1260	0.00	0.00	**0.02**	**0.79**	**0.01**
64	1280	0.00	0.00	**0.01**	**0.85**	**0.02**
65	1300	0.00	0.00	0.00	**0.75**	**0.05**
66	1320	0.00	0.00	0.00	**0.57**	**0.11**
67	1340	0.00	0.00	0.00	**0.42**	**0.23**
68	1360	0.00	0.00	0.00	**0.24**	**0.38**
69	1380	0.00	0.00	0.00	**0.13**	**0.55**
70	1400	0.00	0.00	0.00	**0.07**	**0.63**
71	1420	0.00	0.00	0.00	**0.03**	**0.57**
72	1440	0.00	0.00	0.00	**0.02**	**0.44**

A color code following the scheme in Fig. 5 (peak deconvolution) is used to guide the viewer's eye.

Table S4. Mass composition of the fractions collected in experiment F02

Fraction N°	Volume [ml]	Monomers [mg]	Dimers [mg]	Trimers [mg]	Tetramers [mg]	Pentamers [mg]
25	500	**0.15**	0.00	0.00	0.00	0.00
26	520	**0.35**	0.00	0.00	0.00	0.00
27	540	**0.72**	0.00	0.00	0.00	0.00
28	560	**8.88**	0.00	0.00	0.00	0.00
29	580	**21.44**	0.00	0.00	0.00	0.00
30	600	**29.91**	0.00	0.00	0.00	0.00
31	620	**31.63**	0.00	0.00	0.00	0.00
32	640	**29.01**	0.00	0.00	0.00	0.00
33	660	**25,26**	0.00	0.00	0.00	0.00
34	680	**20,29**	0.00	0.00	0.00	0.00
35	700	**15,54**	**0.08**	0.00	0.00	0.00
36	720	**10,84**	**0.06**	0.00	0.00	0.00
37	740	**7.00**	**0.04**	0.00	0.00	0.00
38	760	**4.03**	**0.04**	0.00	0.00	0.00
39	780	**2.03**	**0.10**	0.00	0.00	0.00
40	800	**1.21**	**0.37**	0.00	0.00	0.00
41	820	**0.39**	**1.92**	0.00	0.00	0.00
42	840	**0.18**	**6.52**	0.00	0.00	0.00
43	860	0.00	**14.43**	0.00	0.00	0.00
44	880	0.00	**20.74**	0.00	0.00	0.00
45	900	0.00	**22.56**	0.00	0.00	0.00
46	920	0.00	**20.97**	0.00	0.00	0.00
47	940	0.00	**17.55**	0.00	0.00	0.00
48	960	0.00	**13.00**	**0.04**	0.00	0.00
49	980	0.00	**8.65**	**0.16**	0.00	0.00
50	1000	0.00	**5.28**	**0.72**	0.00	0.00
51	1020	0.00	**2.85**	**3.10**	0.00	0.00
52	1040	0.00	**1.17**	**7.60**	0.00	0.00
53	1060	0.00	**0.43**	**12.38**	0.00	0.00
54	1080	0.00	0.00	**15.71**	0.00	0.00
55	1100	0.00	0.00	**15.99**	0.00	0.00
56	1120	0.00	0.00	**13.79**	0.00	0.00
57	1140	0.00	0.00	**10.13**	**0.46**	0.00
58	1160	0.00	0.00	**6.73**	**1.83**	0.00
59	1180	0.00	0.00	**3.83**	**4.38**	0.00
60	1200	0.00	0.00	**1.83**	**7.12**	**0.07**

61	1220	0.00	0.00	**1.14**	**8.20**	**0.25**
62	1240	0.00	0.00	**0.45**	**7.90**	**0.84**
63	1260	0.00	0.00	**0.17**	**6.63**	**2.01**
64	1280	0.00	0.00	**0.09**	**4.55**	**3.91**
65	1300	0.00	0.00	0.00	**2.79**	**5.67**
66	1320	0.00	0.00	0.00	**1.83**	**6.21**
67	1340	0.00	0.00	0.00	**0.77**	**5.87**
68	1360	0.00	0.00	0.00	**0.34**	**4.49**
69	1380	0.00	0.00	0.00	**0.21**	**3.06**
70	1400	0.00	0.00	0.00	**0.14**	**1.78**
71	1420	0.00	0.00	0.00	**0.11**	**1.07**

A color code following the scheme in Fig. 5 (peak deconvolution) is used to guide the viewer's eye.
